# The effect of blood flow restriction training on abdominal visceral fat and plasma N-lactoylphenylalanine among adults with obesity

**DOI:** 10.1186/s12902-026-02441-5

**Published:** 2026-07-27

**Authors:** Shuoqi Li, Tiankuo Cui, Shiming Li, Xinyu Zheng

**Affiliations:** 1 School of Sports Science, Nantong University, Nantong, Jiangsu 226019 China; 2 Faculty of Educational Sciences and Technology, Universiti Teknologi Malaysia, Johor Bahru, 81310 Johor Malaysia; 3 Department of Physical Education, Ocean University of China, No.238 Songling Road, Qingdao City, Shandong Province 266100 China

**Keywords:** Blood flow restriction, Moderate-intensity continuous exercise, Obesity, Visceral fat, Multi-omics analysis

## Abstract

**Background:**

Obesity has been designated as one of the diseases that threaten global human health. Blood flow restriction (BFR) exercise may promote the secretion of N-lactoylphenylalanine (Lac-phe) by increasing metabolic stress, and plasma Lac-phe levels may serve as a biomarker associated with fat mass modulation. This study seeks to investigate the effects and mechanisms of moderate-intensity continuous training (MICT) combined with BFR on abdominal visceral adipose tissue (VAT).

**Methods:**

Young adults with obesity were randomly divided into three groups: the non-intervention control group (C group), the MICT intervention group (M group), and the MICT combined with BFR intervention group (B group). After the baseline test, a 12-week exercise intervention was carried out. Before and after the intervention, morning fasting blood samples were collected for the analysis of plasma non - targeted metabolomics and proteomics data, and the body composition, abdominal subcutaneous adipose tissue (SAT), and VAT area were assessed.

**Results:**

After 12 weeks of intervention, the reduction in VAT in the B group was significantly greater than that in the C group. Exploratory multi-omics analyses identified differences between the B group and the M group, with the most prominent enrichment in the glycine, serine and threonine metabolism pathway. Correlation analysis revealed associations between Lac-phe and multiple indicators, including positive correlations with blood lactic acid, glycine and APOA1, and negative correlations with VAT, SAT and leucine (nominal *p* < 0.05).

**Conclusion:**

Compared with traditional MICT, 12-week BFR intervention can significantly reduce abdominal VAT area, and its mechanism may be associated with increased metabolic stress and subsequent secretion of lipolytic hormones. All omics and correlation results are exploratory due to the low sample size and lack of correction for multiple comparisons. Plasma Lac-phe is preliminarily suggested as a candidate biomarker for VAT response to BFR training, which requires validation in larger targeted studies.

**Trial registration:**

ISRCTN registry (Approval number: ISRCTN11246966, http://www.isrctn.org/) on May 8, 2025.

**Clinical trial number:**

This study was prospectively registered in the ISRCTN registry (Approval number: ISRCTN11246966, http://www.isrctn.org/) on May 8, 2025. The first participant was enrolled on May 15, 2025, and the trial intervention was initiated on May 31, 2025, after the completion of all participant enrollment and baseline assessments. The primary outcome prespecified in the registry was the change in abdominal VAT area after 12-week intervention, and the secondary outcomes included changes in SAT area, plasma untargeted/targeted metabolomics and proteomics.

## Introduction

Obesity is a disease that poses a threat to human health worldwide, and an increase in visceral adipose tissue (VAT) is a particularly severe danger. Exercise is an effective way to improve body composition and metabolic health. In recent years, an increasing number of studies have demonstrated that traditional moderate-intensity continuous training (MICT) can improve the body composition of young adults with obesity, especially in terms of reducing the amount of visceral fat. However, the effectiveness of MICT may depend mainly on the amount of exercise a person participates in [1], which may take a considerable amount of time [2]. Considering the difficulties and fears of long-duration exercise among young adults with obesity, there is an urgent need to identify a more time-effective exercise protocol.

Blood flow restriction (BFR) training can induce local hypoxia in working muscles by occluding arterial blood flow with a unique compression device through the proximal external compression section. In addition, BFR can increase metabolic stress in the body, thereby increasing the secretion of lactic acid (LA) and lipolytic hormones [3]. Previous studies on BFR training have focused predominantly on the effect of muscular hypertrophy, but it remains unclear whether it has potential fat-reducing effects.

A study published in Nature in 2022 revealed that exercise can lead to the production of the metabolite N-lactoylphenylalanine (Lac-phe) [4]. Lac-phe is a pseudo dipeptide produced from LA and phenylalanine (Phe) [5]. Lac-phe has been shown to reduce body fat mass in obese mice by suppressing appetite and is regarded as a novel exercise factor [4, 6]. Moreover, Lac-phe is secreted in large amounts during high-intensity exercise and is closely associated with LA levels [7]. Preliminary research by our team [8] demonstrated that a single session of BFR exercise can reduce subjective appetite and influence the secretion of related appetite hormones in young adults with obesity by increasing Lac-phe levels. However, it is still unclear whether BFR exercise can reduce visceral fat by increasing Lac-phe. Therefore, this study further explored the effects and mechanisms of long-term low-intensity MICT combined with BFR on abdominal visceral adipose tissue (VAT) in young adults with obesity via a multiomics approach.

## Methods

### Participants

By distributing promotional flyers and leveraging online platforms, we openly recruited college students from the Ocean University of China. The inclusion criteria were as follows: (1) age: 18–25 years; (2) body fat percentage exceeding 30%; (3) no history of long-term medication administration, smoking, or excessive drinking; and (4) no exercise-related contraindications, such as cardiovascular or cerebrovascular diseases or respiratory system diseases.

### Experimental design

**Fig. 1:**
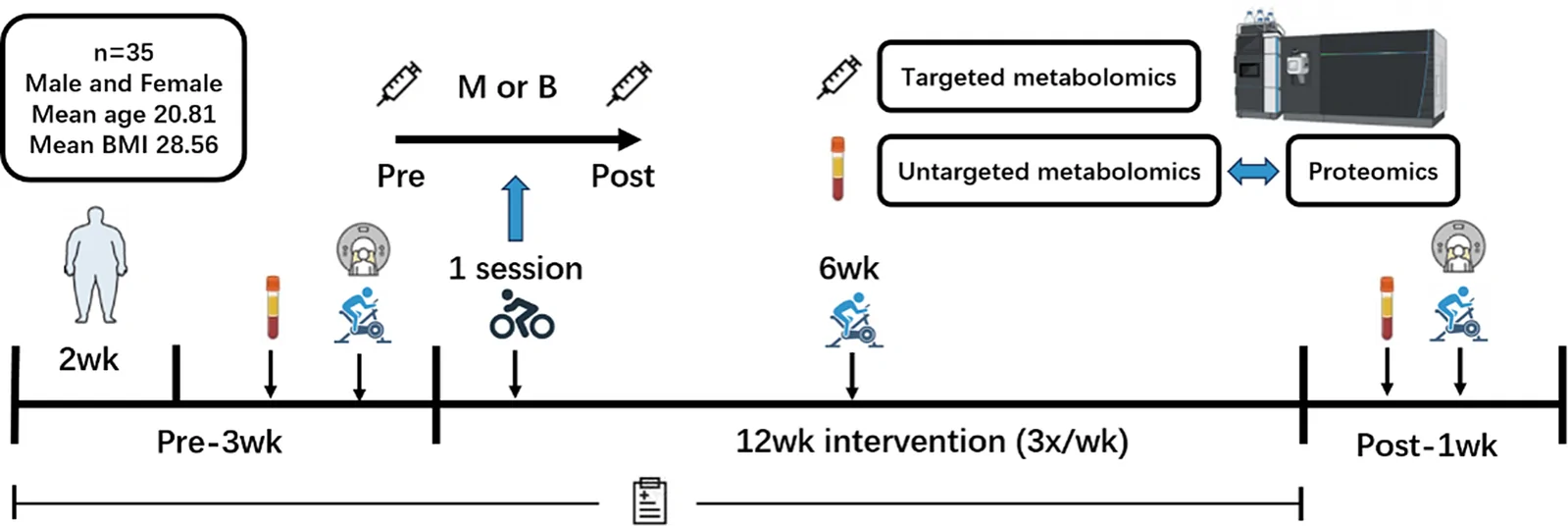
Experimental flowchart. Note:  represents fasting blood sampling in the morning;  represents CT test;  represents maximum oxygen uptake test;  represents acute exercise blood sampling;  represents collection using the Diet and Physical Activity Questionnaire

This study was designed as a single-blind randomized controlled trial. The participants who met the inclusion criteria were randomly assigned to one of three groups: the non-intervention control group (C group), the MICT intervention group (M group), and the MICT combined with BFR intervention group (B group). Once consent was obtained and baseline tests were completed, each participant was assigned to a group, although they were unaware of their group assignment. The participants were randomized by drawing lots. All outcome assessors and laboratory analysts were also blinded to group assignment throughout the study to minimize assessment bias. Participants in the C group were given the same management process and environment as other groups, and added very low-intensity stretching and joint activities. As depicted in Fig. 1, a 12-week exercise intervention was carried out after the baseline test. Fasting blood samples were collected in the morning before and after the intervention for plasma untargeted metabolomics and proteomics analyses. Moreover, the participants’ aerobic capacity, body composition, and abdominal subcutaneous and visceral fat areas were assessed. Daily dietary intake and physical activity were recorded during the 3-week period before the start of the intervention and throughout the 12-week intervention period.

Blood samples were collected before and after the first exercise session to evaluate the changes in the levels of Lac-phe, LA, growth hormone (GH), interleukin-6 (IL-6), epinephrine (EPI), and noradrenaline (NA) following acute exercise. The participants were acquainted with the testing procedure in advance. They entered the laboratory at 8 am after fasting for 12 h and were fed a standardized diet provided by the researchers 60 min prior to exercise. Twenty-four hours prior to the experiment, vigorous exercise, caffeine intake, and alcohol intake were avoided.

### Exercise protocol

The intervention period lasted a total of 12 weeks, with three sessions per week. Researchers continuously monitored the heart rate (HR) and blood oxygen saturation of the participants during exercise to ensure their safety. Initially, participants were required to participate in a 10-minute warm-up activity at an intensity of 50 W on an aerobic power bike. Following warm-up, the participants in all exercise groups continuously exercised at 60% of their maximal oxygen uptake (VO_2max_), maintaining a speed of 60 ± 5 revolutions per minute (RPM), until they completed 200 kJ of mechanical work. After exercise, the participants completed a 10-minute cool-down activity. While following the exercise protocol of the M group, the B group was required to apply a limb occlusive pressure (LOP) of 60% using an inflatable cuff (Ariband, Australia) at the proximal inguinal region of both legs. During exercise, the participants’ rating of perceived exertion (RPE) and HR were measured and recorded every 5 min. The C group did not participate in any exercise intervention throughout the intervention period.

### Limb occlusive pressure test

During the LOP measurements, participants were required to don a BFR inflatable cuff 7 cm in width. The researchers utilized a portable Doppler ultrasound device (Edan, UK) to measure leg artery occlusion pressure. During this measurement, participants were required to lie supine on the bed, with the Doppler probe positioned on the tibial artery to detect the auscultatory pulse. The inflatable cuff was subsequently positioned on the proximal thigh in the inguinal region, and the cuff pressure at which the auscultatory pulse ceased was recorded as the LOP.

### Maximum oxygen uptake test

An incremental load exercise test was carried out using an aerobic power bike (839E, Monark, Sweden). The participants were required to use a specialized gas collection mask. Male and female subjects commenced with initial load intensities of 100 W/50 W, respectively, and the load intensity was increased by 30 W/25 W every 2 min. The speed of the power bike remained at 60 ± 5 RPM until exhaustion. The criteria for exhaustion were as follows: inability to maintain the corresponding speed despite encouragement from the researcher; a respiratory exchange ratio ≥ 1.1; and the emergence of a plateau in oxygen uptake (VO_2_). VO_2_ and HR data during the test were acquired using a gas metabolic analyser (Quark PFT, COSMED, Italy) and a heart rate strap (H12, Polar, Finland). The VO_2_ data from the last 30 s of the final exercise phase were recorded as VO_2max_.

### Body composition test

Body weight and body fat percentage were measured using an Inbody bioelectrical impedance analyser (Korea). The abdominal VAT and SAT areas were quantitatively assessed via computed tomography (CT) (Siemens, Germany). For data collection, the voltage and current were set to 120 kVp and 150 mA, respectively. During the test, the participants were placed in a supine position with their arms relaxed along their sides. Cross-sectional images with a thickness of 5 mm at the umbilical level (approximately between the fourth and fifth lumbar vertebrae) were acquired for evaluation. The attenuation range of the CT images was set from − 190 to -30 HU. Measurements and analyses were conducted by the same blinded technician before and after the intervention.

### Diet and physical activity assessment

On the basis of the previously reported protocol [9], during the 3-week preintervention period and the 12-week intervention period, self-reported physical activity diaries were utilized to estimate habitual energy expenditure. Moreover, Athlete and Public Diet Nutrition Analysis and Management System software was utilized to record the daily diet of each participant for the purpose of estimating calorie intake.

### Blood collection

Blood samples were collected from the elbow vein before and after long-term intervention, as well as immediately before and after a single acute exercise test. A total of 5 mL of venous blood was drawn from the forearm vein and collected in a heparin-coated anticoagulant tube. The blood samples were subsequently centrifuged at 2000 × g for 10 min in a low-temperature centrifuge. The supernatant was aliquoted and stored at -80 °C for subsequent hormone and omics testing and analysis.

### Hormone testing methods

GH (EK1104-48, Lianke Biotech), IL-6 (EK106HS-48, Lianke Biotech), EPI (CSB-E08677h, Cusabio Biotech), and NA (CSB-E07021h, Cusabio Biotech) were tested using enzyme-linked immunosorbent assay (ELISA) before and after acute exercise.

### Untargeted metabolomics method

Untargeted metabolomics allows diverse metabolites in biological samples to be profiled without target preselection and uses techniques such as LC‒MS/GC‒MS to explore overall changes and discover unknown metabolites. The plasma samples collected before and after the 12 weeks of intervention were subjected to untargeted metabolomic analysis using liquid chromatography‒tandem mass spectrometry (LC‒MS/MS) (Thermo Scientific, Waltham, USA) in full-scan mode. The parameters for the electrospray ionization source were as follows: atomizing gas flow rate of 3 L/min; heating gas flow rate of 10 L/min; interface temperature of 500 °C; DL temperature of 250 °C; hot block temperature of 400 °C; and drying gas flow rate of 10 L/min. The raw data obtained were analysed using Compound Discover 3.3 software. With this software, high-quality metabolic signals (S/*N* > 10) in the MS2 spectra were first compared with those in a database and annotated in batches. Metabolic signals that did not match any information in the database were identified by querying MS2 spectral data from the literature or searching other online databases, such as METLIN and MassBank. Compounds for which standards were available were identified by comparing their retention times, accurate m/z values, and fragmentation patterns with those obtained after injection of the standards under similar conditions.

### Targeted metabolomics method

In accordance with previous methods, targeted metabolomics analysis was conducted via LC‒MS/MS in negative ion mode to determine the plasma levels of Lac-phe and La before and after exercise. Approximately 50 µL of each plasma sample was mixed with 250 µL of methanol, vortexed for 30 s, and then centrifuged at 16,000 × g for 20 min at 4 °C. An aliquot of 200 µL of the supernatant was dried under vacuum, and the dried sample was resuspended in 50 µL of 25% ACN. Component separation was performed using an ACQUITY UPLC HSS T3 column (pore size = 1.8 μm, length = 2.1 × 100 mm) (Waters, Milford, USA). The mobile phase consisted of solvents A (6.5 mM aqueous ammonium bicarbonate (NH_4_)HCO_3_) and B (95% methanol in 6.5 mM aqueous (NH_4_)HCO_3_). The instrument was operated in full-scan mode with a resolution of 140,000 and a mass-to-charge range of 70–1050 m/z. The spray voltage was set to 3.00 kV. The flow rates of the nitrogen sheath gas and nitrogen auxiliary gas were fixed at 45 and 10 AU, respectively, while the temperatures of the capillary and auxiliary gas heaters were set to 300 °C and 350 °C, respectively. Parallel reaction monitoring was used to achieve high-resolution spectral resolution (17500) for Lac-phe (m/z = 236.093) via MS/MS with a collision energy of 30 eV. The signal intensity was normalized to the intensity of the internal standard d5-Phe (615870, Merck, Germany), which was added to the extraction solvent to a final concentration of 0.8 µg/mL.

### Proteomics (4D-DIA) method

Approximately 30 µl of sample was thawed on ice, and 270 µl of phosphate buffer solution and 1 mM phenylmethylsulfonyl fluoride were added in sequence. The mixture was shaken at room temperature for 5 min. The total protein concentration was measured via the BCA method. An equivalent amount of protein from each sample was digested with trypsin. Then, 200 µl of 8 M urea was added to the supernatant, which was reduced with 10 mM dithiothreitol at 37 °C for 45 min, followed by alkylation with 50 mM iodoacetamide at room temperature in the dark for 15 min. Four volumes of frozen acetone were added, and the solids were precipitated at -20 °C for 2 h. After centrifugation, the protein precipitate was air dried, resuspended in 200 µl of 25 mM ammonium bicarbonate solution and 3 µl of trypsin, and digested overnight at 37 °C. After digestion, the peptides were desalted using a C18 filter cartridge, dried in a vacuum concentrator, concentrated via vacuum centrifugation, and redissolved in 0.1% formic acid.

### Data analysis

Before and after the intervention, the multiomics data, including the complete metabolite and protein datasets, were uploaded to the MetaboAnalyst 6.0 online platform (https://www.metaboanalyst.ca/). During data preprocessing, missing values were imputed using the NKK (feature-wise) method, the data were filtered on the basis of the interquartile ranges, and the sample data were normalized using logarithmic transformation and Pareto scaling methods. Normality was assessed via the Shapiro-Wilk test. Paired t-tests were used for within-group comparisons before and after intervention. For intergroup comparisons of body composition, abdominal fat areas and VO₂max, analysis of covariance (ANCOVA) was performed with three covariates: baseline values of the corresponding indicator, mean daily calorie intake during the 12-week intervention, and sex. Least significant difference (LSD) post-hoc tests were used for pairwise comparisons after ANCOVA. For other indicators (e.g., exercise intensity parameters, acute hormone responses) with no significant association with dietary intake or sex, one-way ANOVA was used for intergroup comparisons. Considering the exploratory nature of this hypothesis-generating multi-omics study, false discovery rate (FDR) correction was not performed for untargeted metabolomics, proteomics and correlation analyses, as strict multiple testing correction would lead to loss of potential differential molecules. To reduce false-positive risks induced by numerous statistical tests, we combined multiple control measures: differential molecules were screened by the threshold of fold change (FC) ≥ 2.0 or ≤ 0.5 and raw *p* < 0.05 [7, 24]. All omics and correlation results are exploratory. Multivariate statistical analysis was performed via partial least squares discriminant analysis (PLS-DA) to evaluate the differences in the samples before and after intervention and among the groups; the model robustness was validated by R² (0.82–0.91), Q² (0.75–0.84) and 200-times permutation tests. An a priori sample size calculation was performed using G*Power 3.1.9.7 software to determine the required sample size for the primary study endpoint. Based on the previous investigation [12] of exercise-induced VAT reduction in obese young people, the effect size (0.5), a bilateral significance level (α = 0.05) and an expected statistical power (1-β = 0.80) were set. The sample size calculation shows that each group has at least 11 participants (a total of 33 participants) to meet the minimum sample size requirements. Considering that the dropout rate of participants is estimated at 10%, we initially recruited 36 participants. Pearson’s correlation analyses were performed to explore the associations between post-acute exercise changes in Lac-phe levels and other indicators including metabolites, proteins, VAT and SAT after the 12-week intervention. A total of 171 pairwise correlations were tested, based on 19 key indicators: Lac-phe, LA, glycine, APOA1, VAT, SAT, leucine, glutamate, APOE and CRP. The analyses were conducted on pooled exercise groups namely the M group and B group. Statistically significant differences are indicated by *p* < 0.05. The data are presented as the means ± standard deviations (SD).

## Results

### Basic information of the participants

As shown in Table 1, a total of 36 participants were involved in the study, with one person in the M group withdrawing for personal reasons. There were no adverse events throughout the intervention. Before the intervention, there were no significant differences (*p* > 0.05) between the groups in terms of various indicators. Compared with the baseline level, after 12 weeks of intervention, the body fat percentage of M group and B group significantly decreased (*p* < 0.05), and the maximum oxygen uptake significantly increased (*p* < 0.05). C group did not show significant changes in body fat percentage and maximum oxygen uptake. The post-hoc test results show that when the total sample size is 35, Power = 82.27%.

**Table 1:** Basic information and maximal oxygen uptake of participants

Index	C group	M group	B group
Age (y)	21.50 ± 1.73	20.27 ± 1.95	20.17 ± 1.85
Gender (Female: Male)	6: 6	5: 6	6: 6
Height(cm)	170.28 ± 9.84	171.49 ± 12.81	172.52 ± 9.16
Weight (kg)			
Week 1	87.36 ± 20.23	81.64 ± 21.60	84.05 ± 18.38
Week 12	87.58 ± 20.98	79.82 ± 20.56^*^	81.08 ± 17.46^*^
BMI(kg/m^2^)			
Week 1	30.13 ± 4.95	27.76 ± 3.48	28.24 ± 4.05
Week 12	30.19 ± 5.18	27.14 ± 3.20^*^	27.25 ± 3.88^*^
Fat%			
Week 1	37.45 ± 3.73	36.46 ± 3.51	36.18 ± 4.11
Week 12	37.83 ± 3.57	35.51 ± 3.38^#^	34.05 ± 5.01^#^
VO_2max_(ml/min/kg)			
Week 1	32.38 ± 5.20	31.01 ± 3.68	30.64 ± 5.98
Week 6		32.32 ± 3.65^*^	33.20 ± 5.56^*^
Week 12	32.96 ± 5.36	34.70 ± 3.92^*#^	36.08 ± 5.27^*#^

Note: * = A significant difference compared to Week 1, paired sample t-test with *p* < 0.05; # = A significant difference compared to Week 6, paired sample t-test with *p* < 0.05. BMI = Body mass index; Fat% = Body fat percentage; VO_2max_ = Maximal oxygen uptake

### Exercise protocol

The exercise protocol was based on that in a previous study [10]. Table 2 presents the exercise intensity and duration for the M group and B group during the intervention period. Both HR and RPE during exercise were significantly greater in the B group than in the M group (*p* < 0.05).

**Table 2:** Exercise intensity and limb occlusion pressure

Index	Time	M group	B group	MD (95%CI)
Heart rate (beat/min)	Week 1–6	133.85 ± 3.05	144.62 ± 4.79^*^	10.76 (7.24,14.28)
	Week 7–12	131.33 ± 3.05	143.33 ± 4.73^*^	12.00 (8.51,15.49)
RPE	Week 1–6	12.51 ± 0.87	15.18 ± 0.83^*^	2.67 (1.94,3.41)
	Week 7–12	12.75 ± 0.86	15.52 ± 0.71^*^	2.77 (2.09,3.46)
Power (W)	Week 1–6	68.45 ± 18.29	67.92 ± 19.92	-0.54 (-17.17,16.10)
	Week 7–12	75.18 ± 19.77	79.83 ± 23.21	4.65 (-14.13,23.44)
Work (kJ)	Week 1–6	200	200	
	Week 7–12	200	200	
Duration (min)	Week 1–6	52.17 ± 14.68	52.94 ± 14.89	0.77 (-12.07,13.62)
	Week 7–12	47.19 ± 12.32	45.21 ± 13.37	-1.94 (-13.12,9.24)
LOP (mmHg)	Week 1–6		203.67 ± 13.53	
	Week 7–12		200.83 ± 11.88	
60%LOP (mmHg)	Week 1–6		122.20 ± 8.12	
	Week 7–12		120.50 ± 7.13	

Note: * = A significant difference compared to M group, independent sample t-test with *p* < 0.05. RPE = Rating of perceived exertion; LOP = Limb occlusion pressure

### Energy intake and expenditure

Table 3 presents a statistical summary of daily dietary intake and physical activity levels during the initial 3 weeks and the 12-week intervention period. The results indicate that there were no significant differences (*p* > 0.05) in any of the indicators among the groups before and after the intervention.

**Table 3:** Energy expenditure and intake before and during the 12 weeks intervention period

Group	Time	Intake (kcal/d)	Expenditure (kcal/d)
C group	Before	1947.67 ± 150.12	453.17 ± 46.24
	12 Weeks	1964.25 ± 152.70	465.20 ± 64.63
		16.58 (-112.46, 145.48); *p*-value: 0.79	12.03 (-35.84, 60.10); *p*-value: 0.61
M group	Before	1917.80 ± 153.57	506.54 ± 63.43
	12 Weeks	1935.99 ± 154.47	470.23 ± 59.90
		18.19 (-119.43, 155.77); *p*-value: 0.79	-36.31 (-91.34, 18.90); *p*-value: 0.18
B group	Before	1993.95 ± 173.80	464.33 ± 71.09
	12 Weeks	1947.04 ± 143.34	472.64 ± 65.74
		-46.91 (-181.64, 87.50); *p*-value: 0.48	8.31 (-49.60, 66.16); *p*-value: 0.77

### Lac-phe and lipolytic hormone levels

**Fig. 2:**
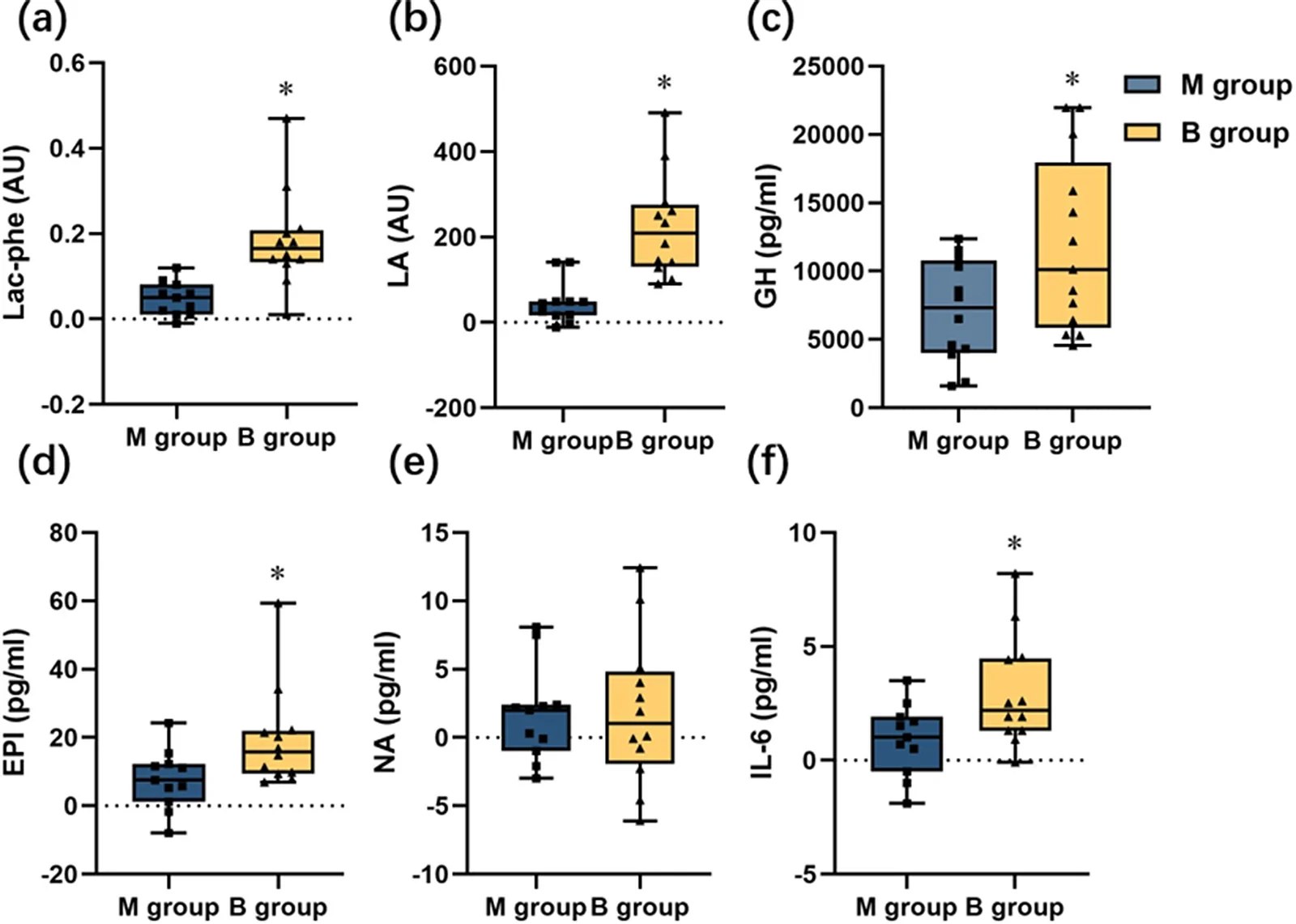
Differences in changes in Lac-phe (**a**), LA (**b**), GH (**c**), EPI (**d**), NA (**e**) and IL-6 (**f**) between M group and B group before and after acute exercise. Note: * = A significant difference compared to M group, independent sample t-test with *p* < 0.05

Figure 2 shows a chart comparing the differences in various blood indicators before and after acute exercise in the M group and B group. The results show that the increases in Lac-phe [mean difference (MD): 0.14 (0.06, 0.21); 0.01], LA [MD: 177.93 (96.14, 259.71); 0.01], GH [MD: 4168.42 (409.33, 7927.51); 0.01], EPI [MD: 7.34 (0.20, 14.47); 0.01], and IL-6 [MD: 1.83 (0.16, 3.49); 0.01] levels in the B group were significantly greater than those in the M group. There was no significant difference in the change in NA levels between the two groups before and after exercise [MD: 2.67 (-6.14, 11.47); 0.56].

### Abdominal fat distribution

**Fig. 3:**
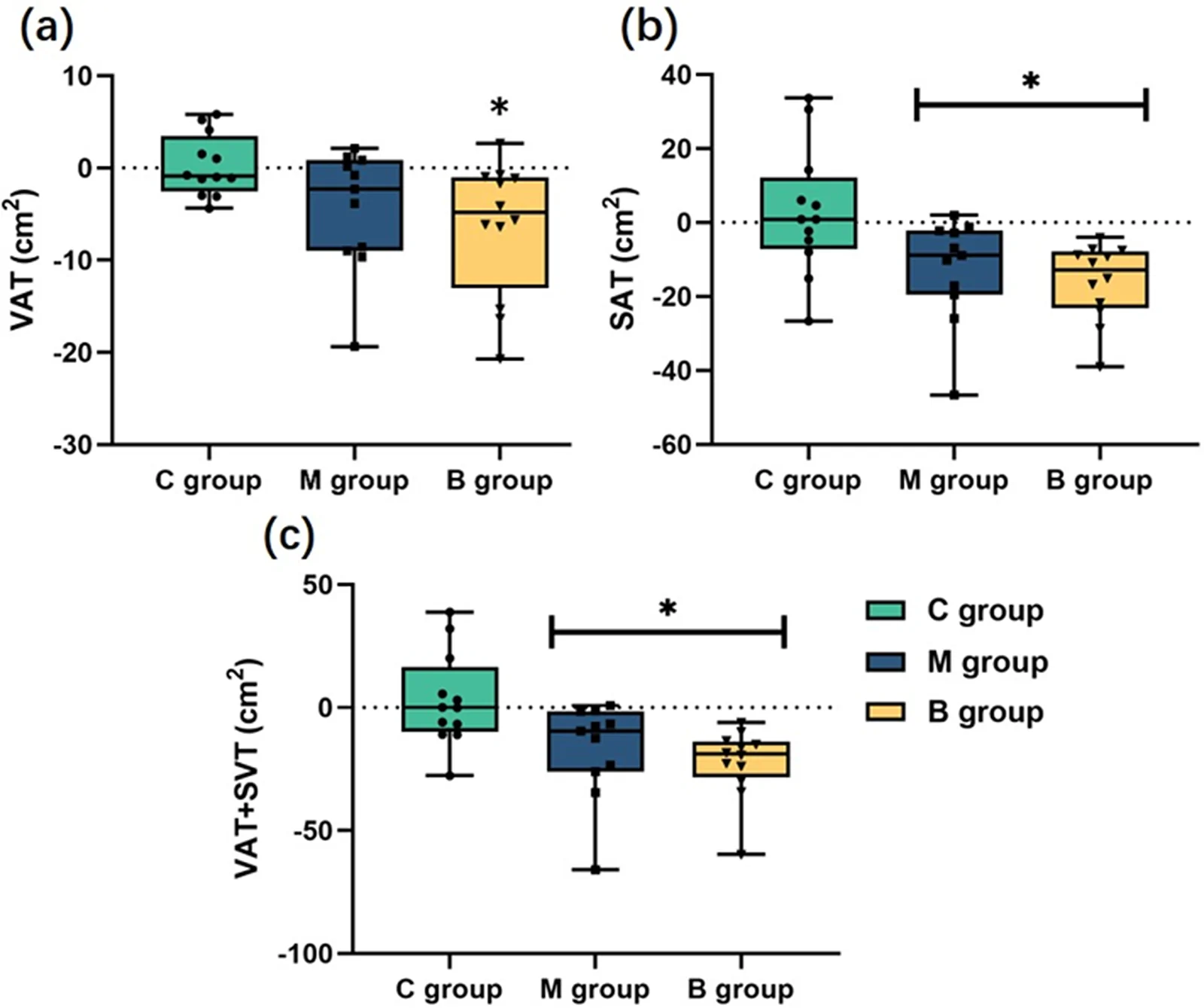
Changes in VAT(**a**), SAT(**b**) and total area (**c**) before and after intervention in each group. Note: * = A significant difference compared to C group, independent sample t-test with *p* < 0.05

Figure 3 illustrates the changes in abdominal VAT and SAT in each group before and after intervention. There were no significant differences in the baseline levels of each indicator among the groups before the intervention. After 12 weeks of intervention, the reductions in SAT and total abdominal fat mass in the B group [MD: -20.77 (-34.62, -6.91); 0.01] and M group [MD: -16.70 (-31.42, -1.97); 0.01] were significantly greater than those in the C group; additionally, the reduction in VAT in the B group was significantly greater than that in the C group [MD: -5.96 (-10.77, -1.14); 0.01], whereas the reductions in VAT in the M group and C group were similar and not significantly different [MD: -4.65 (-9.67, 0.37); 0.08].

### Untargeted metabolomics

**Fig. 4:**
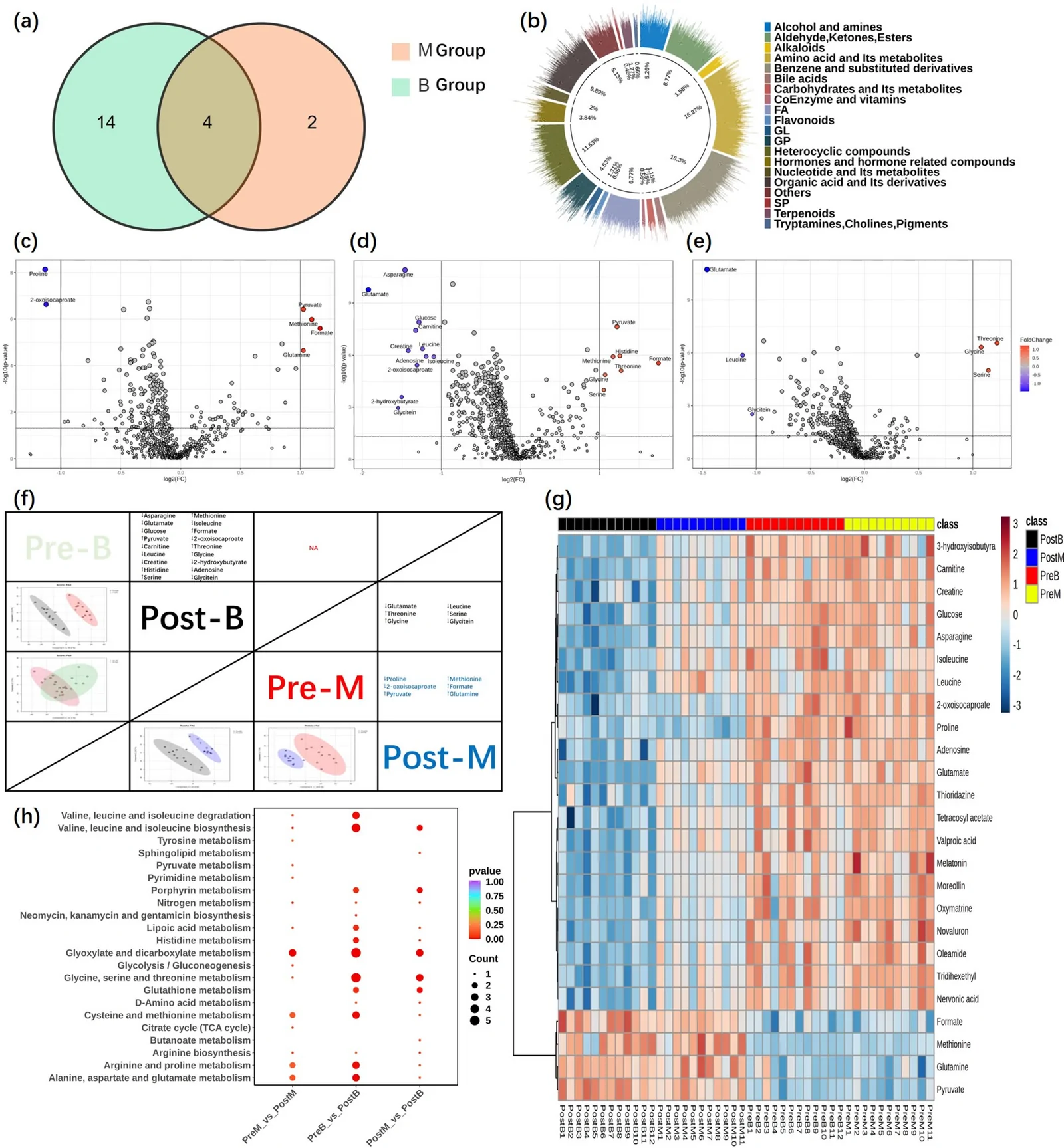
Plasma metabolomics data of M group and B group before and after intervention

All metabolomics results presented below are exploratory, based on nominal p-values without FDR correction. As shown in Fig. 4a, there were 6 differentially abundant metabolites in the M group and 18 in the B group before and after the 12-week intervention (nominal *p* < 0.05, FC ≥ 2.0 or ≤ 0.5). An intersection analysis of the two groups yielded 4 intersecting metabolites. Figure 4b shows a donut chart the main categories of metabolites. A total of 3043 metabolites were detected, including 495 amino acids and their metabolites, 496 benzene and substituted benzene derivatives, 351 heterocyclic compounds and 301 organic acids and their derivatives. Figure 4c shows volcano plots of the differentially abundant metabolites between the M group before and after the intervention, with 4 metabolites increased (pyruvate, methionine, formate, and glutamine) and 2 decreased (proline and 2-oxoisocaproic acid ester). Figure 4d shows the volcano plots of differentially abundant metabolites in the B group pre- vs. post-intervention. Seven metabolites were increased (pyruvic acid, histidine, serine, methionine, formate, threonine, glycine) and 11 decreased (asparagine, glutamate, glucose, carnitine, leucine, creatine, adenosine, isoleucine, 2-oxoisocaproic acid ester, 2-hydroxybutyrate, glycitein). Intergroup comparison identified 3 increased (threonine, glycine, serine) and 3 decreased (glutamate, leucine, glycitein) metabolites in the B group vs. M group (Fig. 4e-f).

### Proteomics

**Fig. 5:**
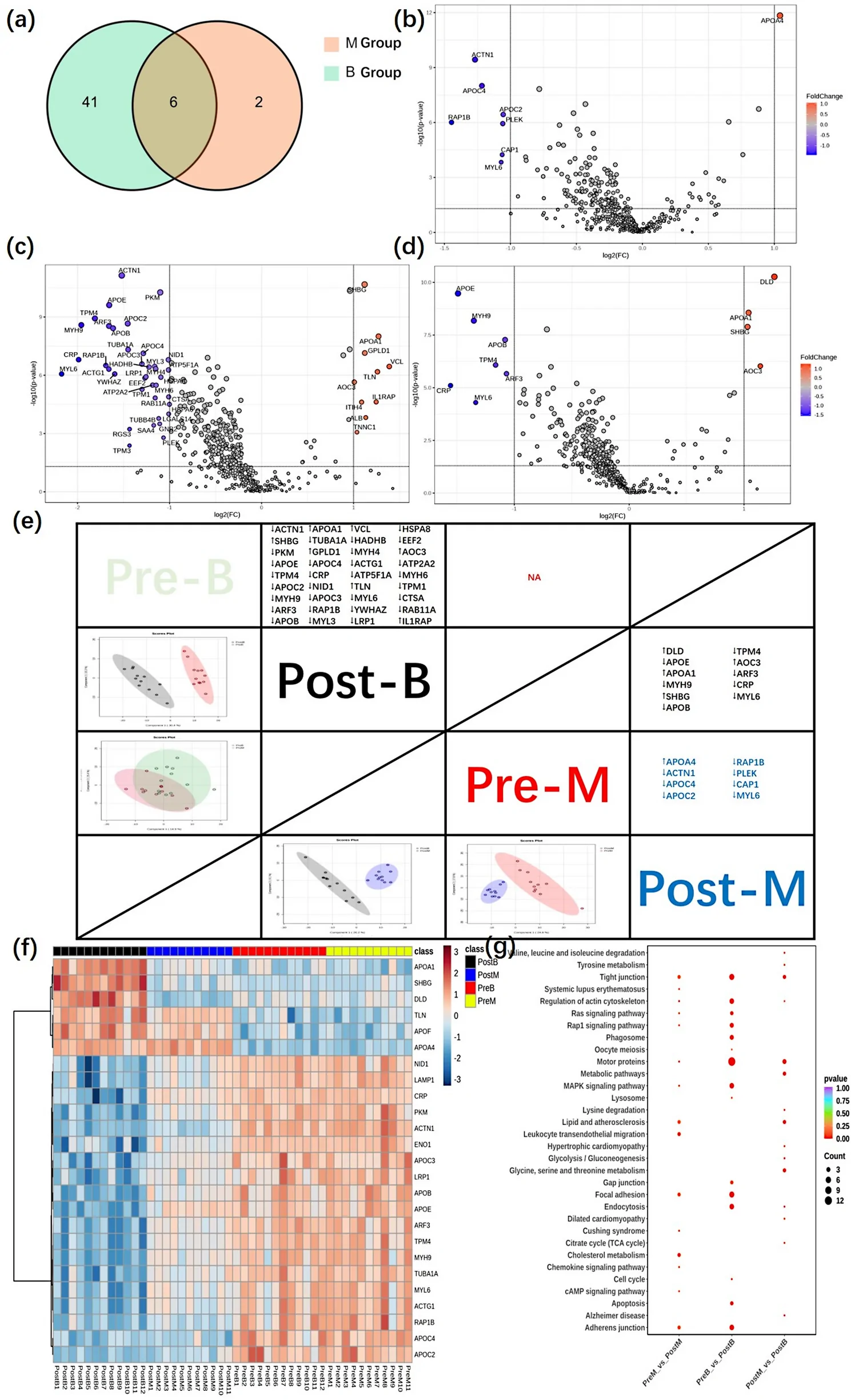
Plasma proteomics data graph of M group and B group before and after intervention

As shown in Fig. 5a, before and after the 12-week intervention, there were 8 differentially expressed proteins in the M group and 47 differentially expressed proteins in the B group. An intersection analysis between the two groups yielded 6 intersecting proteins. Figure 5b shows volcano plots of the differentially expressed proteins in the M group before and after the intervention, with 1 protein upregulated (APOA4) and 7 downregulated (ACTN1, APOC4, APOC2, RAP1B, PLEK, CAP1, and MYL6). Figure 5c shows the volcano plots of the differentially expressed proteins in the B group before and after the intervention, with 10 proteins upregulated (including SHBG, APOA1, GPLD1, and VCL) and 37 downregulated (including ACTN1, PKM, APOE, TPM4, APOC2, MYH9, ARF3, APOB, TUBA1A, APOC4, CRP, NID1, APOC3, RAP1B and MYL3). Figure 5d shows the volcano plots of the differentially expressed proteins in the M group and B group after the intervention, with 4 proteins upregulated (DLD, APOA1, SHBG and AOC3) and 7 downregulated (APOE, MYH9, APOB, TPM4, ARF3, CRP and MYL6). Figure 5e shows the proteomics analysis of the two groups before and after the intervention, which revealed that, with the exception of the data before the intervention, all the other comparison groups exhibited good clustering effects with no obvious intersection, showing the good distinction of the groups. Figure 5f and g shows a heatmap and bubble plot of the enriched proteins in each group before and after the intervention, highlighting that the tight junction, actin, lipid and atherosclerosis, and glycine, serine, and threonine metabolism pathways were the main pathways enriched in the differentially expressed proteins between the B group and the M group after the intervention.

### Combined multiomics analysis

**Fig. 6:**
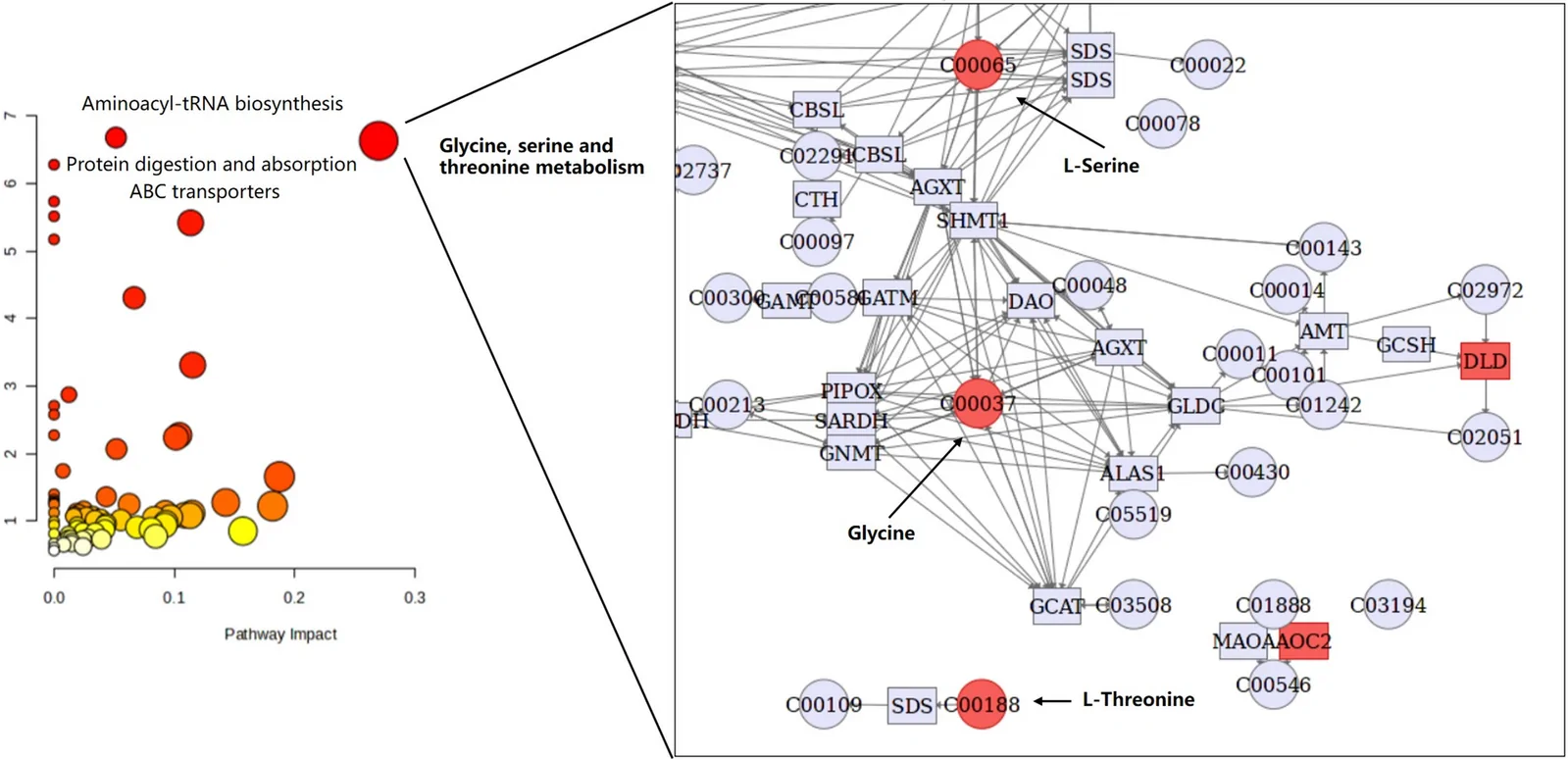
Co-enrichment diagram of metabolism and proteomics

Figure 6 presents the combined enrichment diagram of the metabolomic and proteomic data. The results indicate that pathways such as glycine, serine, and threonine metabolism, aminoacyl-tRNA biosynthesis, protein digestion and absorption, and ABC transporters were the main pathways enriched in the differentially abundant metabolites and differentially expressed proteins between the M group and the B group. Among them, the pathway with the highest value was glycine, serine, and threonine metabolism, which was enriched in three metabolites, serine, glycine, and threonine, and two proteins, DLD and AOC.

**Fig. 7:**
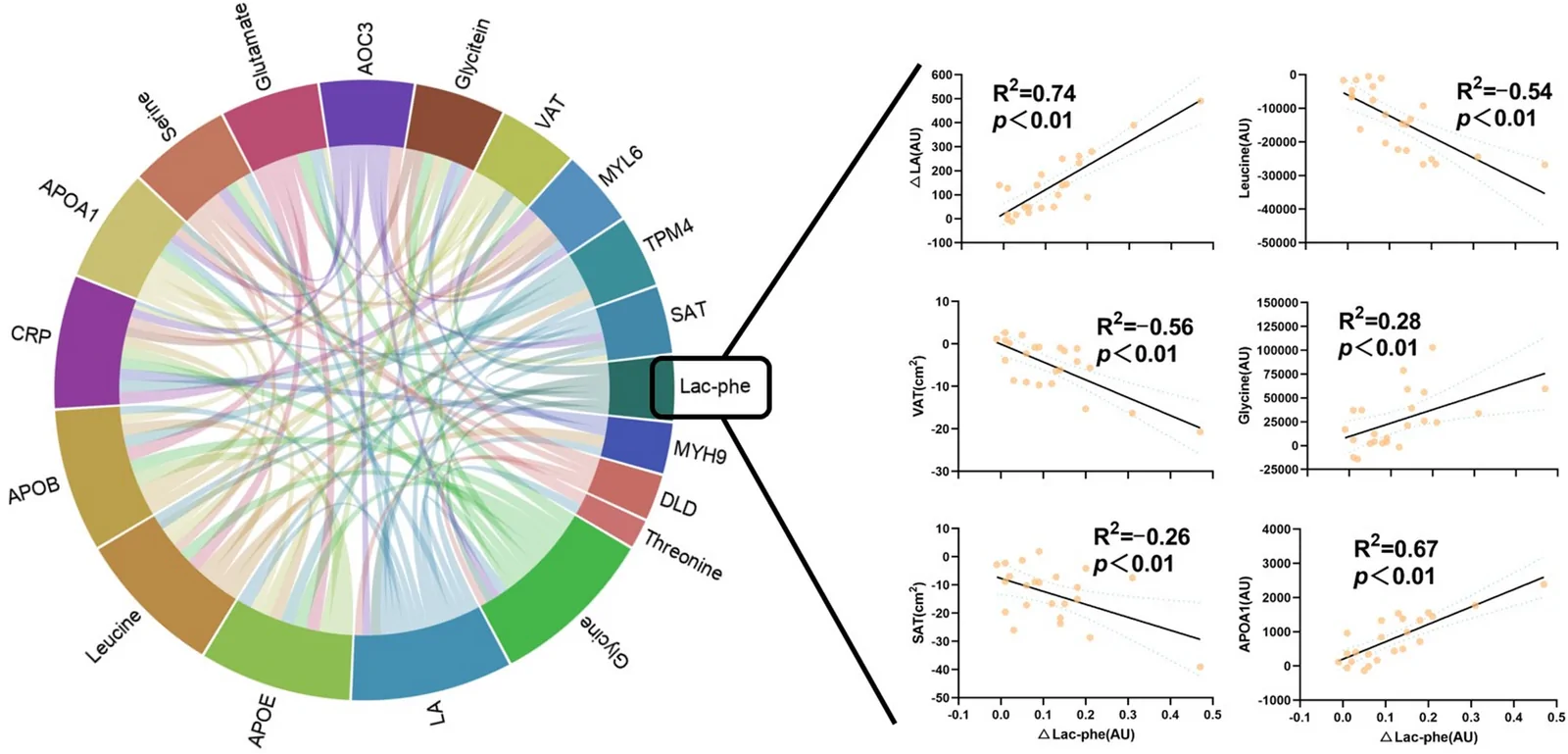
Correlation between Lac-phe and various difference indicators after intervention. Note: Δ represents the difference before and after exercise. Pearson correlation test was used for statistics. Pearson correlations among 19 indicators (171 total pairwise tests; no multiple-testing correction applied; nominal p-values shown)

Figure 7 illustrates the correlation between the differences in the changes in the Lac-phe contents after acute exercise and various other indicators after long-term intervention. A total of 171 pairwise correlations were tested in the M group and B groups. Lac-phe was significantly positively correlated with LA, glycine, and APOA1 and significantly negatively correlated with VAT, SAT, and leucine. Furthermore, VAT was significantly positively correlated with SAT, glutamate, leucine, APOE, and CRP and significantly negatively correlated with glycine and APOA1.

## Discussion

This study investigated the effects and mechanisms of 12 weeks of MICT combined with BFR on abdominal visceral adipose tissue (VAT) in young adults with obesity via a multiomics approach. The results indicated that, in comparison with MICT, BFR exercise significantly elevated postexercise levels of Lac-phe, IL-6, EPI, and GH. Furthermore, long-term BFR intervention led to a more significant reduction in the area of abdominal VAT and subcutaneous adipose tissue (SAT). In addition, the main differentially abundant metabolites between the MICT and BFR intervention groups were glutamate, glycine, leucine, and others; the differentially expressed proteins were DLD, APOE, APOA1, and others; and the main pathway enriched in these factors was glycine, serine, and threonine metabolism. Although there was no decrease in self-reported food intake, the increase in Lac-phe level induced by acute exercise was significantly correlated with postintervention changes in VAT, SAT, glycine, leucine, and APOA1. These results imply that BFR training may promote the reduction of abdominal VAT in two ways: by increasing Lac-phe and lipolytic hormone levels and modulating the plasma levels of glutamate, leucine, glycine, APOE, APOA1, and CRP.

### Exercise protocol

The exercise protocol, which was designed according to a previous study [1], adopted an exercise intensity of 200 kJ and 60% VO_2max_. This MICT protocol has been proven to effectively reduce the body fat percentage in young adults with obesity. The results revealed that the average heart rate during MICT exercise was 133.85 ± 3.05 bpm, which falls within the moderate-intensity range. However, the average heart rate during BFR exercise was significantly greater than that during traditional MICT, which is consistent with previous findings [11].

### The impact of BFR intervention on body composition

In a study by Chen et al. [12], young adults with obesity were subjected to 45 min of aerobic exercise intervention twice a week for 12 weeks with an exercise intensity of 40% VO_2max_. The results indicated that the BFR group achieved a greater reduction in body fat percentage than did the non-BFR group. The lack of a reduction in the body fat percentage in the non-BFR group may be attributed to the low exercise intensity. Although the study investigated the fat-reducing effect of combining BFR with low-intensity training, it did not evaluate the impact of this intervention on VAT. Compared with SAT, VAT poses a particularly severe threat to health and is closely associated with various obesity-related complications [13, 14]. The findings of this study revealed that, despite both exercise groups presenting similar effects on body fat percentage, BFR intervention was more effective in improving VAT than MICT intervention was. Although we did not find significant differences in energy intake or consumption, the average energy intake of Group B seemed to decrease slightly, which may be related to appetite suppression; however, since there was no significant difference, this speculation should be treated with caution. In the study by Kargaran et al., elderly women underwent dual-task training three times a week for 8 weeks with or without BFR at an exercise intensity of 45% heart rate reserve and a BFR stress of 50% LOP. The results showed that the BFR group presented a greater reduction in visceral fat mass than the non-BFR group did. These data support the findings of this study and provide strong evidence for the promotion of visceral fat mass reduction by BFR [15].

### Effects of acute BFR exercise on plasma Lac-phe and lipolytic hormone levels

The mechanism behind the BFR-induced reduction in visceral fat mass may be attributed primarily to the accumulation of LA due to increased metabolic stress following BFR. Lac-phe levels are closely related to LA levels, and BFR can induce an increase in Lac-phe levels. Lac-phe has been proven to be a metabolite that suppresses appetite in humans [4]. Recent publications in Nature Metabolism also indicate that metformin can cause appetite suppression and weight loss by inducing the metabolite Lac-phe [16, 17]. However, these studies did not assess actual food intake after the level of Lac-phe increased in humans. The results of this study show that BFR exercise significantly increased Lac-phe levels; however, although there was a certain decrease in food intake during the intervention period, the difference was not significant. The reason for the lack of a significant difference in self-reported food intake remains unclear, but it is speculated to be a result of the short duration of Lac-phe induction after exercise. Studies have shown that the peak level of Lac-phe after high-intensity exercise occurs one hour after exercise and then persists for three hours before returning to baseline levels. Although high levels of Lac-phe persist longer than LA does after exercise, the time during which exercise affects appetite was very limited throughout the entire intervention period. Therefore, the appetite suppression mediated by Lac-phe after BFR exercise should be considered with caution.

Second, IL-6 is a traditional exercise factor released by skeletal muscle and a key receptor signal that mediates a reduction in visceral fat after exercise. Studies have shown that administering IL-6 receptor antibodies to humans can offset the reduction in visceral fat caused by 12 weeks of exercise intervention without affecting total fat mass [18]. The results of this study indicate that BFR can significantly increase the concentration of plasma IL-6 after exercise, which is consistent with previous findings. Patterson et al. evaluated the changes in blood hormone levels during low-intensity resistance exercise at 20% 1RM in seven elderly individuals, with the BFR group donning an inflatable cuff at 110 mmHg during resistance exercise. The results showed that BFR significantly increased the level of IL-6 after exercise, which was maintained for more than 2 h [19]. Therefore, it is speculated that IL-6 may be one of factors by which BFR exercise reduces visceral fat.

In addition, this study evaluated the levels of GH and EPI after BFR exercise, and the results revealed significant increases in both. BFR during exercise can increase metabolic stress in the body, leading to increases in the secretion of GH and catecholamines during exercise [20, 21]. Catecholamines (such as EPI and NA) are the most important signals regulating fat decomposition and the activity of hormone-sensitive triglyceride lipase (HSL) [22], and fat decomposition during exercise mainly relies on signals from the adrenal gland rather than the sympathetic nervous system [23]. Therefore, the presence of more catecholamines may be more conducive to fat decomposition during exercise. Additionally, studies have shown that visceral fat is more sensitive to lipolytic hormones than is subcutaneous fat [24, 25], which may further promote the decomposition of visceral fat through HSL. In summary, the mechanism by which BFR reduces visceral fat may rely mainly on increases in IL-6, GH, and EPI, while the role of the Lac-phe appetite axis needs to be further determined in subsequent research.

The observed associations between plasma profiles and VAT reduction are mechanistically grounded in the unique physiology of visceral adipose tissue and its systemic crosstalk [26]. Unlike subcutaneous fat, VAT is densely innervated by sympathetic nerves and highly expresses β₃-adrenergic receptors, making it exquisitely sensitive to catecholamine-mediated lipolysis via activation of HSL and adipose triglyceride lipase [21, 23]. BFR-induced metabolic stress amplifies sympathetic outflow, driving preferential triglyceride breakdown in VAT.

For the novel exercise factor Lac-phe, emerging evidence indicates it crosses the blood-brain barrier to modulate hypothalamic energy homeostasis [4]. Beyond appetite suppression, central Lac-phe signaling may enhance sympathetic activity, indirectly promoting VAT lipolysis—consistent with our finding that acute Lac-phe elevation correlates with long-term visceral fat loss. The enriched glycine-serine-threonine pathway further reflects VAT remodeling, as glycine inhibits VAT inflammation and enhances mitochondrial fatty acid oxidation [27]. Importantly, plasma metabolomic and proteomic profiles represent only surrogate markers of systemic metabolic status and cannot definitively reflect intra-tissue molecular changes within VAT itself, as plasma concentrations reflect net release from multiple organs, hepatic clearance and renal excretion. Paired VAT biopsies will be required to characterize tissue-specific mechanisms in future studies.

### Multiomics analysis of the impact of BFR intervention

Consistent with this physiological framework, our exploratory multiomics analysis identified candidate metabolic pathways associated with BFR-induced VAT reduction. The most prominently enriched pathway in both metabolomic and proteomic data was glycine, serine, and threonine metabolism, followed by aminoacyl-tRNA biosynthesis, protein digestion and absorption, and ABC transporters. These pathway enrichment results are based on uncorrected nominal p-values and should be interpreted as hypothesis-generating only. Among them, the glycine, serine, and threonine metabolism pathway is the most significant and has the greatest impact. As shown in Fig. 6, glycine occupies a central position throughout the entire metabolic pathway and correlation analysis revealed a significant negative correlation between glycine and VAT, which is consistent with previous studies [28–30]. Although we cannot infer causality from the correlation between glycine and VAT, a regulatory relationship may exist. Studies have shown that after rats are fed sucrose supplemented with glycine for 4 weeks, the amount of intraperitoneal fat and levels of plasma free fatty acids are significantly reduced and liver mitochondrial respiration is increased. These findings suggest that glycine may reduce VAT accumulation by increasing the rate of fatty acid metabolism [27]. In addition, different exercise regimens have different effects on aerobic capacity, and the current results cannot be used to determine how aerobic capacity affects other parameters.

Although Lac-phe did not significantly affect actual dietary intake, the Lac-phe level may be able to predict the effect of the intervention o.

n VAT. Similar conclusions were also reached in the study by Hoene et al., where 22 young adults with obesity participated in an 8-week aerobic training protocol. The results showed that the level of Lac-phe secreted after acute exercise was closely related to the change in VAT after 8 weeks of aerobic exercise [7]. Independent of its signalling function, Lac-phe may serve as a biomarker to predict an individual’s response to exercise intervention. Future research could enhance the Lac-phe response by optimizing exercise protocols including dietary supplements to improve the effectiveness of exercise interventions.

### Limitations

The present study has several limitations that should be acknowledged when interpreting the results. First, although the sample size was determined via a priori power calculation and provided sufficient statistical power for the primary endpoint, the relatively small sample size (*n* = 35) is underpowered for high-dimensional untargeted metabolomics and proteomics analyses. To avoid losing biologically meaningful signals, no FDR correction was applied to omics or correlation analyses, which increases the risk of false-positive findings. A total of 171 pairwise correlation tests were performed, with an expected 8–9 false-positive associations by chance alone. Thus, all plasma metabolomic and proteomic results reported herein are designated as exploratory and not suitable for definitive statistical inference or causal conclusions. Second, the study population was restricted to young obese college students, with no inclusion of other age groups, or obese populations with comorbidities. This limits the generalizability of our findings to the broader obese population. Third, dietary intake and physical activity were assessed via self-reported diaries and questionnaires, which are inherently prone to recall and reporting bias; in addition, although sex distribution was balanced across groups, sex-stratified analyses were not conducted due to the small sample size, and hormonal status (menstrual cycle phase) was not evaluated, which may represent potential confounding factors for endocrine and metabolic outcomes related to VAT reduction. Finally, omics analyses were only performed on plasma samples, and other tissue metabolomic and proteomic changes were not assessed, which restricts the in-depth mechanistic interpretation of the observed plasma metabolite and protein alterations related to VAT reduction. Future studies with larger sample sizes, diverse study populations, multi-tissue omics profiling, and longer follow-up periods are warranted to validate the exploratory omics findings and confirm the long-term efficacy and underlying mechanisms of BFR intervention for visceral fat reduction.

## Conclusion

Compared with traditional MICT, 12-week BFR intervention can significantly reduce the abdominal VAT area, and its mechanism may be associated with the secretion of lipolytic hormones resulting from increased metabolic stress. All multi-omics results in this study are exploratory observations due to the limited sample size and the absence of FDR correction for multiple comparisons. The primary differential plasma metabolites affected by BFR intervention include glutamate, threonine, and glycine, and the altered plasma proteins include DLD, APOE, and APOA1. Glycine, serine, and threonine metabolism was the most significantly enriched pathway among these differential molecules. Although acute elevation of Lac-phe induced by BFR did not lead to significant changes in dietary intake, this exploratory analysis preliminarily indicates that plasma Lac-phe may serve as a candidate biomarker and potential predictor for visceral fat improvement in response to BFR training. Of note, neither causal nor mediating role of Lac-phe in VAT reduction has been confirmed, and this finding must be validated by subsequent targeted studies with larger sample sizes.

## Data Availability

Data used and/or analysed during the course of this study may be made available by the corresponding author upon reasonable request.

## Abbreviations

BFR: Blood flow restriction
Lac-phe: N-lactoylphenylalanine
MICT: Moderate-intensity continuous training
VAT: Visceral adipose tissue
SAT: Subcutaneous adipose tissue
LA: Lactic acid
Phe: Phenylalanine
GH: Growth hormone
IL-6: Interleukin-6
EPI: Epinephrine
NA: Noradrenaline
HR: Heart rate
VO₂max: Maximal oxygen uptake
LOP: Limb occlusive pressure
RPE: Rating of perceived exertion
VO₂: Oxygen uptake
CT: Computed tomography
ELISA: Enzyme-linked immunosorbent assay
FC: Fold change
MD: Mean difference
APOA4: Apolipoprotein A-IV
ACTN1: Actinin alpha 1
SHBG: Sex Hormone-Binding Globulin
GPLD1: Glycosylphosphatidylinositol-Specific Phospholipase D1
VCL: Vinculin
APOE: Apolipoprotein E
TPM4: Tropomyosin 4
ARF3: ADP-Ribosylation Factor 3
DLD: Dihydrolipoamide Dehydrogenase
AOC3: Amine Oxidase Copper-Containing 3
APOC4: Apolipoprotein C-IV
APOC2: Apolipoprotein C-II
APOC3: Apolipoprotein C-III
APOB: Apolipoprotein B
PLEK: Pleckstrin
CAP1: Cyclase-Associated Actin Cytoskeleton Regulatory Protein 1
MYL3: Myosin Light Chain 3
MYL6: Myosin Light Chain 6
PKM: Pyruvate Kinase Muscle
NID1: Nidogen 1
RAP1B: Ras-Related Protein Rap-1b
MYH9: Myosin Heavy Chain 9
TUBA1A: Tubulin Alpha 1A
CRP: C-reactive Protein

## References

[1] Zhang H, Tong TK, Kong Z, Shi Q, Liu Y, Nie J. Exercise training-induced visceral fat loss in obese women: The role of training intensity and modality. Scand J Med Sci Sports. 2021;31(1):30–43. 10.1111/sms.13803. 32789898

[2] Zouhal H, Lemoine-Morel S, Mathieu ME, Casazza GA, Jabbour G. Catecholamines and Obesity: Effects of Exercise and Training. Sport Med. 2013;43(7):591–600. 10.1007/s40279-013-0039-8. 23613311

[3] Chen Y, Wang J, Li S, Li Y. Acute effects of low load resistance training with blood flow restriction on serum growth hormone, insulin-like growth factor-1, and testosterone in patients with mild to moderate unilateral knee osteoarthritis. Heliyon. 2022;8(10):e11051. 10.1016/j.heliyon.2022.e11051. PMC9586906 36281415

[4] Li VL, He Y, Contrepois K, et al. An exercise-inducible metabolite that suppresses feeding and obesity. Nature. 2022;606(7915):785–90. 10.1038/s41586-022-04828-5. PMC9767481 35705806

[5] Jansen RS, Addie R, Merkx R, et al. N-lactoyl-amino acids are ubiquitous metabolites that originate from CNDP2-mediated reverse proteolysis of lactate and amino acids. Proc Natl Acad Sci U S A. 2015;112(21):6601–6. 10.1073/pnas.1424638112. PMC4450436 25964343

[6] Lund J, Clemmensen C, Schwartz TW. Outrunning obesity with Lac-Phe? Cell Metab. 2022;34(8):1085–7. 10.1016/j.cmet.2022.07.007. 35921816

[7] Hoene M, Zhao X, Machann J, et al. Exercise-induced N-lactoylphenylalanine predicts adipose tissue loss during endurance training in overweight and obese humans. Metabolites. 2022;13(1). 10.3390/metabo13010015. PMC9863672 36676940

[8] Li S, Guo R, Wang J, et al. The effect of blood flow restriction exercise on N-lactoylphenylalanine and appetite regulation in obese adults: a cross-design study. Front Endocrinol (Lausanne). 2023;14:1289574. 10.3389/fendo.2023.1289574. PMC10728722 38116312

[9] Chow BC, Li S, Zhu X, et al. Effects of descending or ascending stair exercise on body composition, insulin sensitivity, and inflammatory markers in young Chinese women with obesity: A randomized controlled trial. J Sports Sci. 2021;39(5):496–502. 10.1080/02640414.2020.1829362. 33012244

[10] Zhu X, Jiao J, Liu Y, Li H, Zhang H. The release of lipolytic hormones during various high-intensity interval and moderate-intensity continuous training regimens and their effects on fat loss. J Sport Sci Med. 2024;1559–70. 10.52082/jssm.2024.559. PMC11366854 39228779

[11] Bennett H, Slattery F. Effects of Blood Flow Restriction Training on Aerobic Capacity and Performance. J Strength Cond Res. 2019;33(2):572–83. 10.1519/JSC.0000000000002963. 30531417

[12] Chen Y, Ma C, Wang J, Gu Y, Gao Y. Effects of 40% of Maximum Oxygen Uptake Intensity Cycling Combined with Blood Flow Restriction Training on Body Composition and Serum Biomarkers of Chinese College Students with Obesity. Int J Environ Res Public Health. 2021;19(1):168. 10.3390/ijerph19010168. PMC8750492 35010428

[13] Fukuda S, Hirata A, Nishizawa H, et al. Systemic arteriosclerosis and eating behavior in Japanese type 2 diabetic patients with visceral fat accumulation. Cardiovasc Diabetol. 2015;14(1):8. 10.1186/s12933-015-0174-7. PMC4301666 25592402

[14] Ko YH, Wong TC, Hsu YY, Kuo KL, Yang SH. The Correlation Between Body Fat, Visceral Fat, and Nonalcoholic Fatty Liver Disease. Metab Syndr Relat Disord. 2017;15(6):304–11. 10.1089/met.2017.0001. 28481662

[15] Kargaran A, Abedinpour A, Saadatmehr Z, Yaali R, Amani-Shalamzari S, Gahreman D. Effects of dual-task training with blood flow restriction on cognitive functions, muscle quality, and circulatory biomarkers in elderly women. Physiol Behav. 2021;239:113500. 10.1016/j.physbeh.2021.113500. 34174326

[16] Scott B, Day EA, O’Brien KL, et al. Metformin and feeding increase levels of the appetite-suppressing metabolite Lac-Phe in humans. Nat Metab. 2024;6(4):651–8. 10.1038/s42255-024-01018-7. PMC11052712 38499765

[17] Xiao S, Li VL, Lyu X, et al. Lac-Phe mediates the effects of metformin on food intake and body weight. Nat Metab. 2024;6(4):659–69. 10.1038/s42255-024-00999-9. PMC11062621 38499766

[18] Wedell-Neergaard AS, Lang Lehrskov L, Christensen RH, et al. Exercise-Induced Changes in Visceral Adipose Tissue Mass Are Regulated by IL-6 Signaling: A Randomized Controlled Trial. Cell Metab. 2019;29(4):844–e8553. 10.1016/j.cmet.2018.12.007. 30595477

[19] Patterson SD, Leggate M, Nimmo MA, Ferguson RA. Circulating hormone and cytokine response to low-load resistance training with blood flow restriction in older men. Eur J Appl Physiol. 2013;113(3):713–9. 10.1007/s00421-012-2479-5. 22922803

[20] Shimizu R, Hotta K, Yamamoto S, et al. Low-intensity resistance training with blood flow restriction improves vascular endothelial function and peripheral blood circulation in healthy elderly people. Eur J Appl Physiol. 2016;116(4):749–57. 10.1007/s00421-016-3328-8. 26822582

[21] Delfan M, Vahed A, Bishop DJ, et al. Effects of two workload-matched high intensity interval training protocols on regulatory factors associated with mitochondrial biogenesis in the soleus muscle of diabetic rats. Front Physiol. 2022;13:927969. 10.3389/fphys.2022.927969. PMC9541894 36213227

[22] Thompson D, Karpe F, Lafontan M, Frayn K. Physical Activity and Exercise in the Regulation of Human Adipose Tissue Physiology. Physiol Rev. 2012;92(1):157–91. 10.1152/physrev.00012.2011. 22298655

[23] Stallknecht B, Lorentsen J, Enevoldsen LH, et al. Role of the sympathoadrenergic system in adipose tissue metabolism during exercise in humans. J Physiol. 2001;536(1):283–94. 10.1111/j.1469-7793.2001.00283.x. PMC2278837 11579176

[24] Arner P. Catecholamine-induced lipolysis in obesity. Int J Obes. 1999;23(S1):S10–3. 10.1038/sj.ijo.0800789. 10193856

[25] Saeidi A, Saei MA, Mohammadi B, et al. Supplementation with spinach-derived thylakoid augments the benefits of high intensity training on adipokines, insulin resistance and lipid profiles in males with obesity. Front Endocrinol (Lausanne). 2023;14:1141796. 10.3389/fendo.2023.1141796. PMC10422041 37576981

[26] Delfan M, Saeidi A, Supriya R, et al. Enhancing cardiometabolic health: unveiling the synergistic effects of high-intensity interval training with spirulina supplementation on selected adipokines, insulin resistance, and anthropometric indices in obese males. Nutr Metab (Lond). 2024;21(1):11. 10.1186/s12986-024-00785-0. PMC10921712 38454429

[27] El Hafidi M, Pérez I, Zamora J, Soto V, Carvajal-Sandoval G, Baños G. Glycine intake decreases plasma free fatty acids, adipose cell size, and blood pressure in sucrose-fed rats. Am J Physiol Regul Integr Comp Physiol. 2004;287(6):R1387–93. 10.1152/ajpregu.00159.2004. 15331379

[28] Tran AM, Wander PL, Thomas MK, et al. Plasma amino acid profile, a biomarker for visceral adipose tissue that can substitute for waist circumference in Japanese Americans. Obes Res Clin Pract. 2021;15(6):557–63. 10.1016/j.orcp.2021.10.007. PMC8629946 34782257

[29] Takashina C, Tsujino I, Watanabe T, et al. Associations among the plasma amino acid profile, obesity, and glucose metabolism in Japanese adults with normal glucose tolerance. Nutr Metab (Lond). 2016;13:5. 10.1186/s12986-015-0059-5. PMC4717594 26788116

[30] Saeidi A, Hackney AC, Tayebi SM, Ahmadian M, Zouhal H, Diabetes. Insulin Resistance, Fetuin-B and Exercise Training. Ann Appl Sport Sci. 2019;7(2):1–2. 10.29252/aassjournal.7.2.1. PMC6639044 31321377

